# Potential Pharmacological Applications of *Nigella* Seeds with a Focus on *Nigella sativa* and Its Constituents against Chronic Inflammatory Diseases: Progress and Future Opportunities

**DOI:** 10.3390/plants12223829

**Published:** 2023-11-11

**Authors:** Shadma Wahab, Abdulrhman Alsayari

**Affiliations:** Department of Pharmacognosy, College of Pharmacy, King Khalid University, Abha 62529, Saudi Arabia; alsayari@kku.edu.sa

**Keywords:** *Nigella sativa*, phytochemistry, bioactive molecules, chronic inflammatory diseases, toxicology

## Abstract

The leading cause of death worldwide has been identified as chronic illnesses, according to the World Health Organization (WHO). Chronic inflammatory conditions such as asthma, cancer, diabetes, heart disease, and obesity account for three out of every five deaths. Although many people benefit from using nonsteroidal anti-inflammatory medicines (NSAIDs) for pain and inflammation relief, there are significant adverse effects to using these medications. Medicinal plants possess anti-inflammatory properties with minimal or no side effects. *Nigella sativa* (NS), also known as black cumin, is one of the plants used in traditional medicine the most. Many studies on the NS have shown that their therapeutic properties are attributed to the seed, oil, and secondary metabolites. This plant has been studied extensively and has many medical uses, such as anti-inflammatory. NS or its phytochemical compounds, such as thymoquinone, can cause cell apoptosis via oxidative stress, block efflux pumps, enhance membrane permeability, and exert potent biocidal effects. Notwithstanding the extensively documented anti-inflammatory effectiveness observed in the experimental model, the precise mechanisms underlying its anti-inflammatory effects in diverse chronic inflammatory diseases and its multi-targeting characteristics remain largely unexplored. This review examines NS or its secondary metabolites, a valuable source for the therapeutic development of chronic inflammatory diseases. Most clinical studies were done for diabetes and cardiovascular disease; therefore, more studies are required to examine the NS extracts and phytoconstituents to treat cancer, obesity, diabetes, asthma, neurological disorders, and COVID-19. This study will be a significant resource for clinicians and biologists seeking a pharmaceutical solution for inflammatory diseases.

## 1. Introduction

The World Health Organization (WHO) has identified chronic illnesses as the leading cause of death worldwide. Pro and anti-inflammatory endogenous mediators work in concert to eliminate harmful stimuli through the protective biological processes known as inflammatory responses. Chronic inflammatory illnesses, such as asthma, cancer, diabetes, heart disease, and obesity, account for three out of every five deaths [1]. Herbs as a therapeutic intervention for various ailments may be traced back to ancient periods predating recorded history. These drugs have been used to treat and prevent a wide variety of illnesses, including persistent inflammatory disorders. Although many people benefit from using nonsteroidal anti-inflammatory medicines (NSAIDs) for pain and inflammation relief, there are significant drawbacks to using these medications. There are few to no negative consequences from using medicinal herbs for their anti-inflammatory properties [2]. The usage of medicinal plants has increased despite the introduction of pharmacological drugs for treating chronic disorders. Numerous chronic illnesses have emerged due to the rapid changes in human lifestyle and diet that have taken place during the last century. Natural drug use is again a hot subject, as synthetic medication effectiveness declines and new risks emerge. Therefore, researching phytotherapy to treat chronic illnesses may pay off handsomely in terms of potential sources of medicinal plants essential to disease prevention, and whose use and promotion align with all currently practiced preventive methods.

Although many ancient practices have died out in modern Western societies, some are still used by alternative medicine advocates or as complementary treatments. One of the plants used in traditional medicine to control chronic inflammatory ailments is *Nigella sativa* (NS). NS, also called black cumin, black seed, black caraway, fennel flower, Roman coriander or kalonji. This annual plant of the Ranunculus family (Ranunculaceae) is grown for its pungent seeds, which are used as a spice and in herbal medicine. The plant in question, belonging to the Ranunculaceae family, is an annual species cultivated primarily for its aromatic seeds. These seeds are highly valued for their culinary applications as a spice and their use in herbal medicine [3]. Black cumin seeds and their oil are prominent in Ayurveda and traditional Islamic medicine as therapeutic agents for treating several ailments, including chronic illnesses [4]. Thymoquinone (TQ), the most prevalent quinine compound, is a phytoconstituent found in black cumin. TQ possess valuable medicinal properties such as antimicrobial, anti-inflammatory, anti-oxidative, hypolipidemic, hypoglycemic, and bronchodilatory effects, as well as being used for the treatment of metabolic syndrome and auto-immune disorders [5]. Clinical evidence supports the seeds’ antiparasitic, antibacterial, and antifungal activities. Additionally, several animal experiments have shown the ability of these seeds to suppress cancer growth [6]. Anti-inflammatory properties of NS and its secondary metabolites were reported in some inflammation-based models, including asthma, cancer, rheumatoid arthritis, and colitis [7,8]. NS has shown inconsistent results on oxidative stress and inflammation biomarkers in several clinical studies [9,10]. Notwithstanding the extensively documented anti-inflammatory effectiveness observed in the experimental model, the precise mechanisms underlying its anti-inflammatory effects in diverse chronic inflammatory diseases and its multi-targeting characteristics remain largely unexplored. Thus, this review explored NS and its secondary metabolites, a valuable source for the therapeutic development of chronic inflammatory diseases. This study will be a significant resource for clinicians and biologists seeking a pharmaceutical solution for inflammatory diseases.

## 2. The Methodology of the Literature Review

A comprehensive and systematic data search was used to conduct a literature survey. We have searched the data using multiple electronic databases, such as Science Direct (http://www.sciencedirect.com/, accessed on 2 July 2023), PubMed (http://www.ncbi.nlm.nih.gov/pubmed, accessed on 28 July 2023), Web of Science (https://apps.webofknowledge.com/, accessed on 10 August 2023), Scopus (http://www.scopus.com/, accessed on 17 August 2023), and also search by Google Scholar, as well as various books and theses/dissertations. The keywords used to explore the literature for this review were “*Nigella sativa*”, “Black cumin”, “black seed”, “kalonji”, “Antioxidant”; “anti-inflammatory”, “Immunomodulatory”, “Anticancer activity”, “Thymoquinone”, “Antidiabetic activity”, “cardiovascular disease”, “Neurological disorder”, “Obesity”, “Asthma”, and “COVID-19”. Phrases such as the “pharmacological activity of *Nigella sativa* in different diseases” and a combination of phrases were used via electronic search engines. Figures have been drawn with the help of BioRender. We have listed all the relevant research papers from all the existing references and searched for suitable information for this review.

## 3. Global Distribution

People have used this plant for over a thousand years as a medicine. Evidence of the oldest cultivation of NS has been discovered by archaeologists, with dating indicating its presence as early as the 2nd century BCE [11]. *Nigella* is derived from the Latin word Niger, which means “black”. The word “nigellus” denotes a shade of black or dark, explicitly referring to the color of the seed coat. The cultivation of black cumin is seen in several regions throughout Asia, Africa, Europe, and the Americas. NS is growing wild in Turkey, Iraq, Iran, Syria, and Africa [12]. The species is also grown commercially in India [13] and other parts of Southern Asia, Pakistan [14], Iran [15], Western Asia [16], Iraq [16], Israel [17], Jordan [18], Syria [19], Lebanon [20], Turkey [21], Yemen [21], Sudan [22], Northern Africa [22], Egypt [16], Tunisia [16], Ethiopia and Eastern Africa [23]. The primary nations engaged in the production of the commodity are India, Bangladesh, Sri Lanka, Pakistan, Afghanistan, Iraq, Iran, Syria, Ethiopia, and Turkey. Black cumin’s natural average productivity has been reported to be 0.79 tons/ha. In 2018, the black cumin oil market was valued at over 15 million USD; substantial growth is anticipated by 2025 [24]. India cultivates this plant on a large scale due to its heavy consumption in medicine. Annual quantities of black cumin are estimated to be traded in India for more than 100 metric tons. According to Gashaw’s (2020) findings, the average yield in Ethiopia was recorded at 0.64 tons per hectare, a significantly lower figure compared to other nations known for their high agricultural productivity [23]. In 2015, Ermias et al. said the low production was because the cultivars were not very productive [25]. In 2008, Hammo said that it was because the farmers were not using good agronomic practices [26].

### 3.1. Traditional Uses of Nigella

Evidence shows that it was cultivated, used in cooking, and even used as medicine in Mesopotamia from the late 3rd millennium BCE to the late 1st millennium BCE [27]. NS is described in Cuneiform tablets discovered at excavation sites in ancient Assyria (modern-day Turkey, Syria, Iraq, and Iran) as a divine herb that can be used to treat cases of “ghost possession” by fumigating an area with a mixture prepared from NS seed [27,28]. The significance of NS may be assessed based on the observable exportation of this plant from the Mediterranean region. The archaeological findings discovered in the Roman quarries of Mons Claudianus and Mons Porphyrites dating back to the 2nd century provide compelling evidence for the existence of a trade network involving the transportation of *Nigella* seeds from the Mediterranean region to other regions in Western Europe [29,30]. The production of mineralized NS seeds in Germany provides evidence to support the hypothesis of the historical trading of *Nigella* in this geographical area. Dioscorides, a Greek pharmacobotanist, mentions “the black seeds” of a plant called melanthion (NS) in his book “De Materia Medica”, which was published between CE 40–90. He describes its use in foods like pickles and bread and as a treatment for headaches, skin problems, toothaches, leprosy, roundworms, sight imperfections, breathing ailments, urination difficulties, menstrual problems, etc. [31]. This wonder herb has been used in folkloric traditional medicine for thousands of years, long before the development of modern medicine. Unani, Tibb-e-Nabwi, Siddha, Ayurveda, Greek, Roman, Malay, and Jewish texts are some of the many ancient cultures whose written traditional medicinal texts mention this plant [32]. *Nigella* seeds, also known as black seed and black cumin, have been widely used as medicinal remedies throughout many Abrahamic civilizations. The black seed is widely acknowledged for its notable therapeutic attributes, being considered a panacea for all ailments except for mortality [33,34,35]. In tibb-e-nabawi or Prophetic medicine, NS is used expertly [34,35]. *Nigella* is referenced in the Bible on four occasions, including the New and Old Testaments [22].

**Figure 1:**
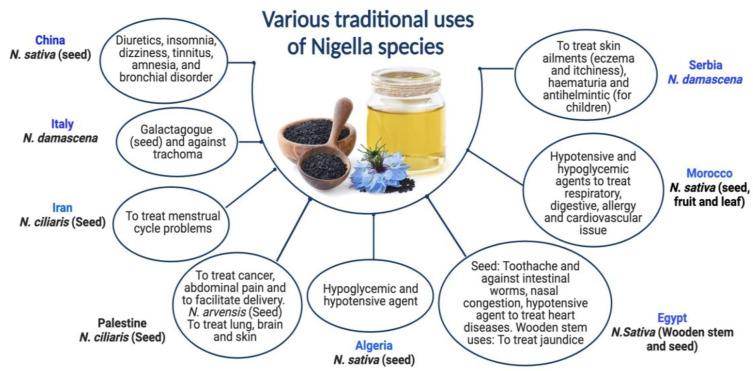
Various traditional uses of *Nigella* species.

*N. ciliaris* and *N. damascena* species of *Nigella* have been shown to provide various health benefits. Although *N. damascena* has been used since the Bronze Age in Central Europe, its ethnobotanical significance cannot be conclusively established. Its origins are still unknown; it has never been seen growing wild in Central Europe [36]. *N. damascena* seeds are a galactagogue in traditional Sicilian medicine [37]. In Serbian mediaeval medicine, this plant is also a helmintic agent to cure skin conditions like hematuria [38]. Italy and Tunisia traditionally use *N. damascena* for treating trachoma [39]. In addition to being used as an herbal cure, *N. damascena* is a condiment in several places [36], including Morocco [40]. *N. ciliaris* seeds are used in the traditional medicine of Palestine and Iran to cure menstrual cycle-related issues, ease childbirth, and relieve stomach discomfort [41,42]. The dried blossoms of *N. arvensis* are used to make winter tea in the Turkish city of Meriç Town [43]. In Iran (Kerman), however, a mixture of *N. arvensis* seed flour and other seeds is administered to enhance male potency and increase memory and intelligence [43,44]. Both *Nigella* species’ seeds are used for treating cancer in Palestine [45,46]. Formulation of black cumin essential oil nanoemulsions (BCO-NE) using different ratios of essential oil with canola and flax seed oils (ripening inhibitors) were formulated and stabilized with octenyl succinic anhydride (OSA) modified waxy maize starch for the antimicrobial use [47]. Pioglitazone is incorporated into a nanoemulsion formulation prepared with NSO to boost the action of Pioglitazone [48]. Numerous individuals use the oil to treat skin disorders like eczema, psoriasis, and blisters. Black seed oil combined with beeswax is also used as a moisturizer, anti-wrinkle agent, burn salve, and treatment for skin infections and joint discomfort. Various traditional uses of *Nigella* species are exhibited in Figure 1.

### 3.2. Usefulness of NS in Religious and Medical Scripts

**Figure 2:**
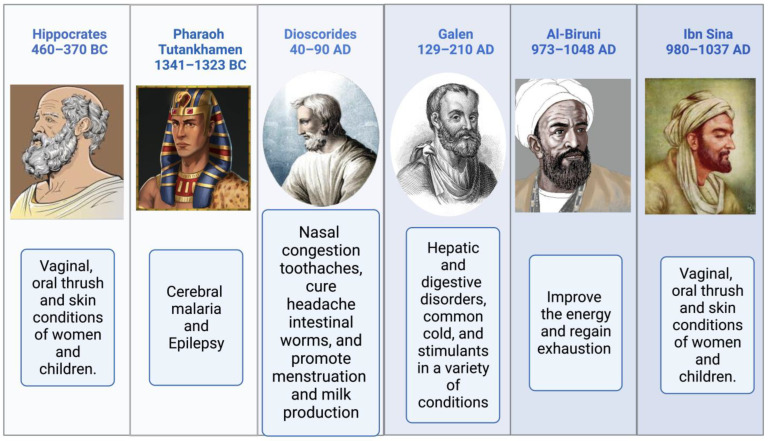
The health benefits of *Nigella sativa* recommended by various scholars.

Religious texts have mentioned the names of medicinal plants, their preparation, and their significance [49,50]. The Prophetic Medicine and the Bible, two essential works from the Ancient and New Testaments, refer to NS (Isaiah 28:25:27 and Matthew 23:23). NS is mentioned more often than any other plant in the Bible. It is used for both birth control and cancer therapy [51]. According to Hippocrates, Dioscorides, and Pliny, NS is the medicinal “black cumin” mentioned in the Bible. It is also referred to as Melanthion and Gith [52]. Prophet Muhammed (Peace Be Upon Him), NS seeds may cure all ailments and diseases except death (Al-Bhukhaari, 5688). Ibn Sina, well-known in the West as Avicenna, and who wrote the renowned book “The Canon of Medicine,” advised about using NS. It increases the body’s vitality and aids in the body’s recovery from exhaustion and depression. Pedanius Dioscorides described utilizing NS seeds for culinary and medicinal uses, including treating eye disorders, toothaches, and leprosy, increasing urination, and repelling snakes [4]. Black seeds and honey syrup were a traditional medicine of the time of Nabi-e-Akram (Peace Be Upon Him), as attested to in the writings of Seerah. Reviewing the relationship between sickness treatment and the meals that the Prophet Muhammad (PBUH) advocated in light of contemporary superfoods [53,54]. Suhar Bakht identified NS, which he dubbed hab-i-Sajzi, or Sigzi grains. The health benefits of *Nigella sativa* were recommended by various scholars, as shown in Figure 2.

## 4. *Nigella sativa*, the Plant, and Its Phytoconstituents

**Figure 3:**
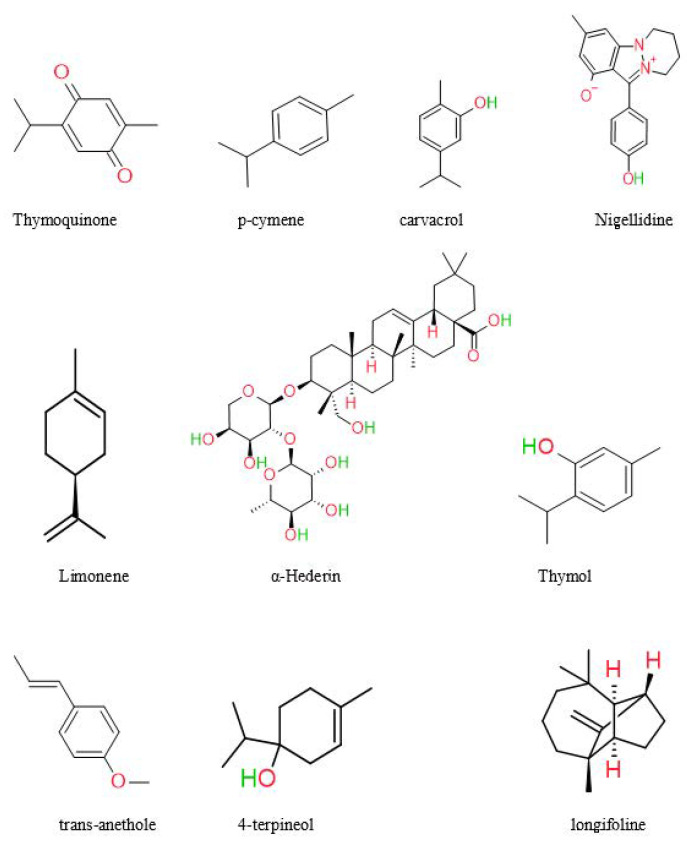
Chemical structures of some main constituents of *Nigella sativa* seeds.

In recent years, significant advancements have been achieved in phytochemistry, and health supplements have gained prominence. The medicinal plant is called a chemical manufacturing industry, since it produces a variety of bioactive molecules such as alkaloids, saponins, oleoresins, resins, glycosides, sesquiterpene lactones, and oils [55]. NS’s most abundant chemical components are thymoquinone, α-phellandrene, thymol, proteins, oleic acid, and carbohydrates [7,56]. Previous research has isolated and characterized the primary components of black seeds, including palmitic acid, oleic acid, linoleic acid, and trans-anethole [57]. A study by Kumar et al. found Quiones (thymohydroquinone, thymoquinone, dithymoquinone) and phenolics [58]. Black seed oil was analyzed by Harzallah et al. and yielded 48 unique chemicals, the majority of which were thymoquinone [59]. According to another research, NS seeds contained various chemicals, most of which were monoterpene hydrocarbons. The constituents of NS seed are 0.4–2.5% essential oil, saponin, alkaloids, and 36–38% fixed oils [60]. Unlike most oils, fixed oil contains the unusual fatty acids C20:2arachidic and eicosadienoic [61]. Burits and Bucar et al. analyzed the essential oil using GC-MS. The major components were characterized as thymoquinone (27.8–57.0%), carvacrol (5.8–11.6%), ρ-cymene (7.1–15.5%), trans-anethole (0.25–2.3%), longifoline (1.0–8.0%), and 4-terpineol (2.0–6.6%). Dithymoquinone is easily formed when thymoquinone dimerizes [62]. The chemical structures of some main constituents of *Nigella sativa* seeds are shown in Figure 3.

## 5. Pharmacological Activities of *Nigella sativa*

Numerous pharmacological effects have been observed for the most widely used traditional medicinal herb, *Nigella sativa.* Thymoquinone, one of the bioactive compounds found in NS, can cause oxidative stress-induced cell apoptosis, enhance membrane permeability, obstruct efflux pumps, and exert potent biocidal effects.

### 5.1. Antioxidant and Anti-Inflammatory

Inflammatory responses are biological processes that protect us from harmful stimuli by coordinating the production of pro- and anti-inflammatory endogenous mediators [63]. NS is a free radical scavenger and improves the action of antioxidant enzymes (glutathione peroxidase, catalase, and glutathione-S-transferase). Thymoquinone’s anti-inflammatory effects were primarily seen in models of inflammation caused by rheumatoid arthritis, colitis, cancer, and asthma [7]. It has been shown to have an anticancer effect via modulating several molecular targets, including p53, p73, STAT3, PTEN, PPAR-g, caspase activation, and reactive oxygen species (ROS) [64]. *Nigella sativa* seed essential oil was tested for its potential antioxidant properties. An examination was conducted in this study using two TLC screening methods. The results of the study have shown that thymoquinone and the components trans-anethole, carvacrol, and 4-terpineol have antioxidant potential [64].

Regarding managing hyperglycemia and boosting beta cell production in the pancreas, NS is superior to other plant species, thanks to its potent antioxidant and antidiabetic characteristics [65]. Several human clinical studies have produced conflicting findings on the impact of NS on oxidative stress and inflammation indicators. Five different methanolic fractions on guinea pigs were investigated to identify the main constituents of the methanolic extract of NS. All the methanolic fractions had significant relaxant effects, and the 20% fractions from NS were more than that of theophylline at the used concentrations [66]. *Nigella sativa* seed oil is widely used in the Mediterranean region for its anti-inflammatory properties [67]. Both NS oil and thymoquinone are effective anti-inflammatory agents, reducing the production of inflammatory mediators, including prostaglandins and leukotrienes, in animal models of diseases such as colitis, encephalomyelitis, oedema, peritonitis, and arthritis [68]. At a 4 mL/kg/day dose, NS oil suppressed NO and IL-4 production in rats when given orally for 31 days [69]. Clinical studies have also shown NS’s anti-inflammatory properties, such as when the oil was administered to older people with osteoarthritis [70]. NS, for instance, may be helpful as a topical therapy for psoriasis. A little research has shown the topical benefits of NS oil. NS, for example, may be beneficial as a topical therapy for psoriasis [71]. Indomethacin and nanoparticles derived from NS essential oil have been demonstrated to have anti-inflammatory effects [72]. A study examined the anti-inflammatory and antioxidant activities of the oil extracted from seeds of *NS* and its biological activity.

### 5.2. Immunomodulatory Effects of Nigella sativa

Modifying the immune response by controlling communication between its many parts-for example, between neutrophils and macrophages or T and B cells-is known as immunomodulation. Immunomodulators can stimulate or repress the immune system, aiding immunological function [73]. The research on the immunomodulatory properties of NS seed extracts, fixed oil, and essential oils that have been published so far is reviewed [74]. It was investigated in vitro how NS seeds and their soluble fractions affected the reactivity of lymphocytes to various mitogens and the phagocytic activity of polymorphonuclear leukocytes. It was observed that NS stimulates the lymphocyte response to combined allogeneic cells. When cultured with pooled allogeneic cells or without any additional stimulator, NS increased the production of interleukin-3 by human lymphocytes. Interleukin-1 was found to be elevated in response to NS, indicating an influence on macrophages. However, when *Staphylococcus aureus* was utilized, no impact of NS or its fractions was seen on bacterial phagocytosis or killing, suggesting that the reduction in chemiluminescence activity in the presence of NS is unrelated to the bactericidal activity [75,76]. An increasing body of experimental research indicates that NS and its active components have powerful and positive immunomodulatory effects. NS has been demonstrated to include elements that stimulate T cell-mediated immune responses and inhibit B cell-mediated immunological responses [77,78]. More research is needed to confirm previous findings and uncover novel immunotherapeutic properties of this promising medicinal herb, NS, because few studies have focused on examining NS’s potential immunomodulatory and immunopharmacological effects and its active ingredients.

### 5.3. Anticancer Activity

The widespread prevalence of cancer makes it a top global health concern [79,80]. We must simultaneously address many global health issues to combat this threat. High death and morbidity rates demonstrate the ineffectiveness of clinical cancer rehabilitation, which includes immunosuppression, chemotherapy, radiation, and surgery. It has increased the need to develop new, more reliable methods of cancer therapy and prevention [81,82,83]. Herbal therapeutics are thus reborn through dietary supplements and botanical preparations. Unique chemical compounds with therapeutic potential are abundant in these medicinal plants [84,85,86,87]. Secondary metabolites are the bioactive substances found in plants and are the building blocks of phytochemicals [88,89,90].

**Figure 4:**
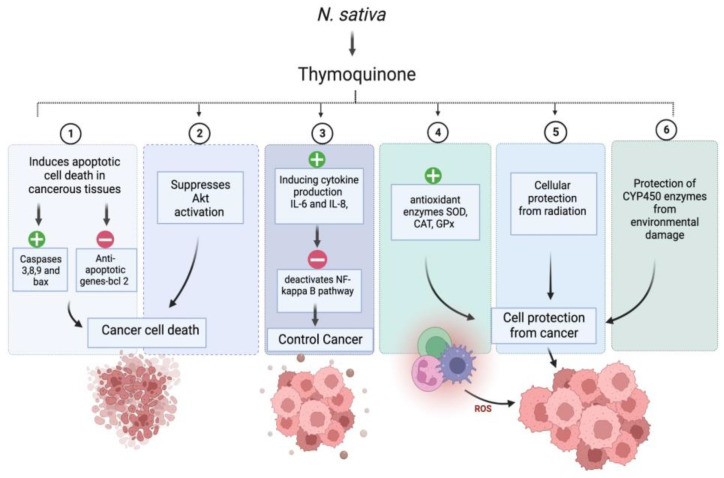
Possible anticancer mechanism of action of thymoquinone (TQ). “+” Means increase and “−” means decrease.

Interestingly, around 60% of anticancer medications have a natural origin [91]. We have compiled a summary of the many anticancer actions linked to NS in this section. Numerous bioactive compounds have been identified in NS seeds. The seeds comprise fixed and essential oils, alkaloids, saponin, and proteins [92,93]. NS’s critical bioactive component, thymoquinone (TQ), is responsible for the plant’s positive anticancer effect. In-vitro and in-vivo studies have demonstrated that TQ has decisive anticancer and antiproliferative activities against liver, blood, respiratory tract, kidney, colon, and prostate cancers. Combination cancer treatment with NS extract or TQ has been shown to reduce the toxicity of traditional cancer medications, while improving the quality of life for people with advanced cancer [94]. The anticancer activity of (R)-limonene was shown to be mediated by the process of apoptosis and the modification of polyamine metabolism. The compound limonene can trigger apoptosis in LS174T colon cancer cells via a mechanism that involves the mitochondria [95]. Researchers discovered that α-hederin caused a dose- and time-dependent rise in the death of murine leukaemia P388 cells. α-hederin has been shown to exhibit strong antitumor activity in vivo [96]. The efficacy of derivatives derived from TQ-bearing terpene-terminated 6-alkyl residues was evaluated in 518A2 melanoma and HL-60 cells. They discovered that the derivatives cause a slight rise in reactive oxygen species, a drop in mitochondrial membrane potential, and apoptosis linked to DNA laddering [97]. It was discovered that NS extracts, both aqueous and alcohol-based, were successful in vitro in deactivating MCF-7 breast cancer cells [98]. TQ has anti-neoplastic properties and promotes apoptosis in the HCT116 colon cancer cell line [99]. The study conducted by Salim et al. demonstrates the ability of the volatile oil of NS to inhibit colon carcinogenesis in rats at the post-initiation stage, without any observable adverse effects [100]. TQ was potent against the SW-626 colon cancer cells, and the results were similar to 5-fluorouracil in action on HT-29 cells [101]. Chehl et al. also suggested TQ as a new way to stop pro-inflammatory pathways. This idea is promising because it combines ways to control inflammation and help cells die [102]. The anticancer activity of TQ is attributed to potent antioxidant properties in normal cells, and prevents the formation of lipid peroxidation and radicals. TQ acts as a prooxidant, increasing ROS production and causing cancer cells to die after oxidative damage. TQ causes cancer cells to undergo apoptosis by upregulating the transcription factor p53, which promotes apoptosis. Upregulation of the proapoptotic p53 transcription factor is how TQ causes cancer cells to apoptosis. TQ is a cytoprotective molecule that mitigates the harmful effects of chemotherapy on healthy cells without interfering with the therapeutic effects of the drugs themselves in the fight against cancer. It stops cancer cells from dividing and halts the process of angiogenesis [94]. Figure 4. exhibits the possible anticancer mechanism of action of thymoquinone (TQ).

### 5.4. Antidiabetic Activity

Diabetes is a chronic disease caused by the pancreas’ inability to produce or properly use insulin. Patients with diabetes mellitus often turn to herbal remedies for relief. Dyslipidemia, elevated oxidative stress, and changes in the body’s antioxidant defense system may all be associated with diabetic complications [103]. Worldwide, 451 million people between the ages of 18 and 99 years have diabetes. By 2045, their numbers were predicted to rise to 693 million. Nearly half of all patients with diabetes (49.5%) are thought to remain undiagnosed. In addition, it was predicted that over 21.3 million live births to women were impacted by some hyperglycemia in pregnancy, and it was expected that 374 million individuals had impaired glucose tolerance (IGT). The prevalence of diabetes-related complications was estimated to range from 20 to 90.5% across several studies [104]. Although there is currently no cure for diabetes mellitus, it may be controlled with medications like insulin and dietary changes. Alternative treatment might come from using medicinal herbs [105].

**Figure 5:**
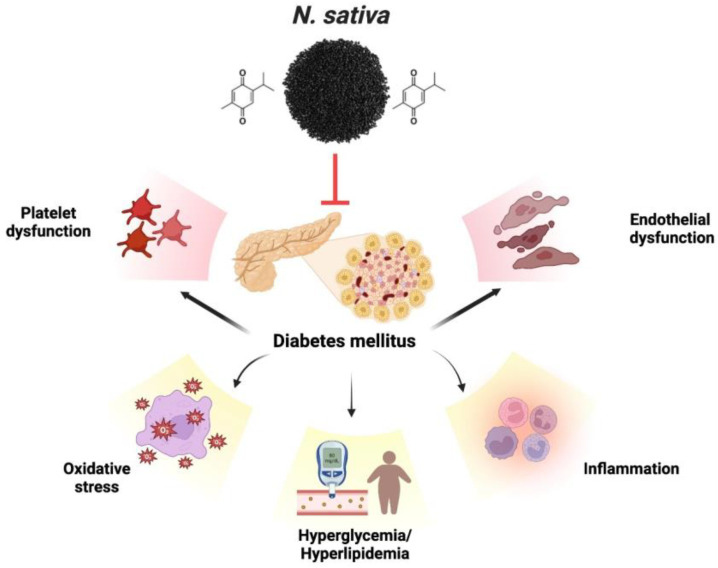
Various complications associated with diabetes.

The therapeutic promise is associated with various medicinal plants and their isolated constituents. Diagnosing the antidiabetic characteristics of food items supplemented with medicinal plants often involves measuring the inhibition of α-glucosidase and α-amylase enzymes. The enzyme α-amylase breaks down carbs into simpler disaccharides, while the enzyme α-glucosidase converts the simpler disaccharides into the more easily absorbed glucose [106,107]. This disease can be treated safely and effectively with natural compounds that inhibit these enzymes [108,109]. Diabetes-induced endothelial dysfunction is protected by thymoquinone. Endothelial dysfunction caused by diabetes is mitigated by thymoquinone. Numerous mechanisms are employed to achieve this, including the decrease in inflammatory and apoptotic markers, enhancement of hyperlipidemia, hyperglycemia, and antioxidant function, inhibition of platelet aggregation, and regulation of gene expression related to VCAM-1, eNOS, and LOX-1, which is implicated in endothelial dysfunction. TQ also inhibits the expression and secretion of specific cytokines, including interleukin-1β, MCP-1, NF-κB, TNF-α, and Cox-2, resulting in an anti-inflammatory effect [110]. Patients with type 2 diabetes were given NS seeds as an adjunct treatment to their anti-diabetes drugs [111]. TQ is one of the principal bioactive chemicals shown to have a defensive effect against diabetes, and it is mainly responsible for the therapeutic actions of NS [112]. Previous research has shown that TQ significantly lowers fasting blood glucose (FBG) and significantly boost rats’ insulin levels [113]. The results of the scrutiny on the effects of *NS* extract on diabetes demonstrate that *NS* extract decreases blood glucose and lipid levels in diabetes. The present portion of the reviews indicates that NS and TQ protect against endothelial dysfunction brought on by diabetes, thanks to their antidiabetic, antioxidant, anti-thrombotic, and gene-regulatory activities. Figure 5 exhibits the various complications associated with diabetes.

### 5.5. Cardiovascular Disease (CVD)

An acute myocardial infarction is consistently ranked as one of the leading causes of death worldwide. Clinical investigators investigated the possible impact of delivering cells to the chemically wounded heart. C-reactive proteins (CRP) are produced in the blood due to inflammation, and their concentration increases as inflammation worsens. Advanced coronary artery inflammation is caused by a pocket of fatty, soft plaque that accumulates and seeps into the arty channel. Rehabilitative methods for the heart include antioxidants, plant life, and physical activity [1]. More people in middle-income nations die from cardiovascular disease than in high-income countries, says the World Health Organization. CVDs are a group of disorders in the heart and blood vessels responsible for the death of approximately 23.6 million people worldwide [114]. People with risk factors like diabetes, high cholesterol, or high blood pressure need more intensive medical and psychological support [115].

One of the most crucial protective factors against CVDs may be eating a diet low in salt, free sugar, and fat, and high in natural plant products [116]. One of NS’s active components, TQ, has been shown to have several biological benefits. Anticancer, antidiabetic, anti-inflammatory, hypolipidemic, and other therapeutic uses of TQ have been established. Flavonoid-rich plants have been utilized for centuries to treat various conditions [117,118]. Due to their antioxidant, anti-inflammatory, and vasodilatory properties, flavonoids are gaining much ground in contemporary pharmacology as a viable therapy for CVDs [119]. TQ contains several bioactive substances [120].

**Figure 6:**
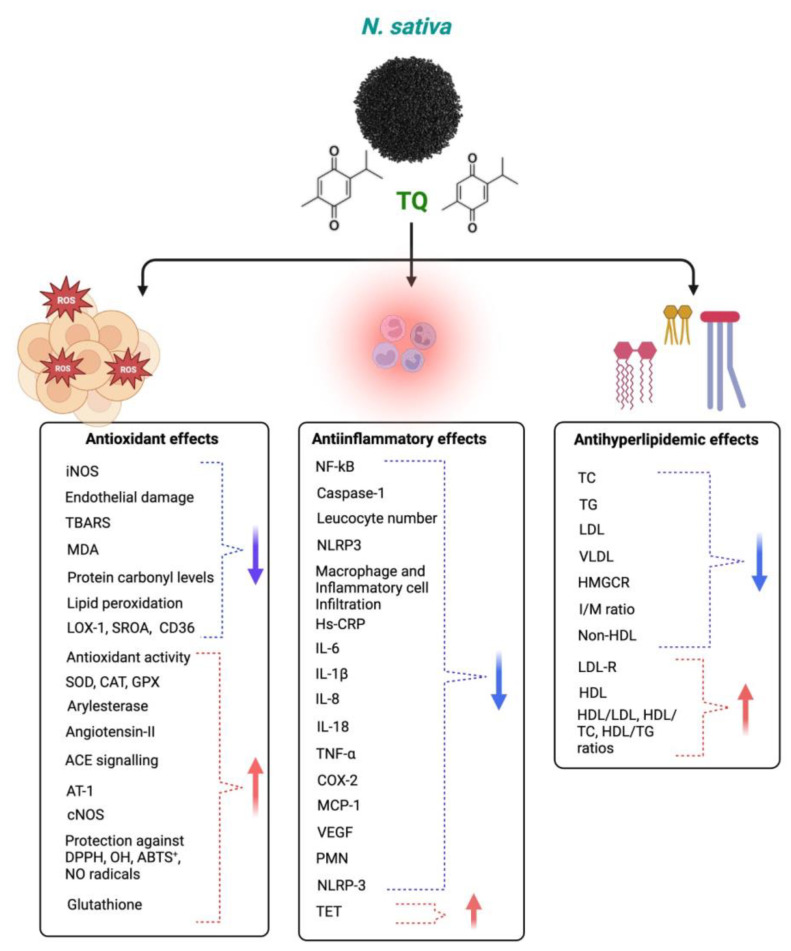
Different mechanisms of *Nigella sativa* and its phytoconstituents for antioxidant, anti-inflammatory, and antihyperlipidemic effects. The blue arrows indicate decreased effects and red arrows indicate increased effects following NS administration.

TQ protects against coronary artery diseases, diabetes, hypertension, inflammation, apoptosis, and oxidative stress. TQ’s anti-inflammatory and antioxidant activities may cause its clinical effect against various diseases [121]. TQ’s antioxidant impact is linked to its scavenging ability against reactive oxygen species (ROS), whereas its anti-inflammatory effect is linked to its inhibitory effects on 5-lipoxygenase and cyclooxygenase [122]. TQ also forms glutathione-dihydro-TQ when combined with glutathione (GSH), NADPH, and NADH; this compound is effective against free radicals [123]. It has been noted that TQ has protective properties for treating and inhibiting CVDs [124]. This section demonstrates how TQ may treat cardiovascular disorders by reducing oxidative and inflammatory reactions. TQ has anti-inflammatory and antioxidant properties, which it uses to combat the many disorders that arise from an imbalance of inflammation and oxidative stress. Although TQ has been found to have cardioprotective benefits in vivo and in vitro settings, the same cannot be said for clinical studies, and more safety evaluations are required to identify the harmful qualities of TQ in people. Additionally, further research is needed to verify its traditional usage as a therapy for cardiovascular disorders. Different mechanisms of actions of *Nigella sativa* and its phytoconstituents for antioxidant, anti-inflammatory, and antihyperlipidemic effects are shown in Figure 6.

### 5.6. Neurological Disorder

One of the worst outcomes of becoming older is neurodegenerative illnesses, which are caused by damage to the nervous system and abnormal biochemical processes in the body. Neuroprotective and neurotrophic effects are at the forefront of current neuroscience and improve brain health. The elderly are particularly vulnerable to neurodegenerative diseases. Neurological disorders are depression, insomnia, and neurodegenerative diseases; common pathological conditions are neurogenesis impairment, neurotrophic factor deficiency, and oxidative disorder stress. Damage to neurons is exacerbated when microglia and astrocytes are activated in response to oxidative stress, increasing the production of pro-inflammatory mediators. Neuronal injury and degeneration result from producing inflammatory cytokines, creating amyloid plaques and neurofibrillary tangles (NFTs) [1]. Many neurodegenerative diseases have no known cure, and the commercial medications available to treat them all have significant drawbacks. Scientists are thus looking for effective medicines with little adverse effects. Researchers are interested in various phytochemicals because of their potential therapeutic benefits and low risk of negative consequences.

There are many different types of herb plants, and NS is only one of them. Many illnesses, including inflammation, oxidative stress, bacterial/fungal infection, and neurological disorders, have been researched concerning the chemical components of plants. In this section of the review article, we have examined the role of *NS* and its secondary metabolites against neurodegenerative diseases. Ayurveda also recommends the extensive use of the black seeds. It aids in the healing of a wide variety of conditions, including those manifesting in the kidneys, the digestive system, the liver, the eyes, the heart, and the nervous system [125].

Numerous investigators have elucidated the actions and impacts of NS on the central nervous system. Alzheimer’s disease (AD) and Parkinson’s disease (PD) are two examples of neurodegenerative illnesses; NS is known to protect against neurotoxicity and cytotoxicity in such cases [126,127,128]. Amyloid β protein (Aβ) is the main component of neuritic plaques in Alzheimer’s disease (AD), and its accumulation has been considered the molecular driver of Alzheimer’s pathogenesis and progression. Aβ has been the prime target for the development of AD therapy [129]. Additionally, it has been shown that NS seed extracts, both aqueous and methanolic, have sedative solid and depressive effects on the central nervous system; in addition to having analgesic properties, diseases like Alzheimer’s cause gradual brain shrinkage and the buildup of senile plaques in the cortex. Amyloid-beta (Aβ) is a peptide with a molecular weight of 4.2 kD, and there is pathological evidence that it aggregates in the central nervous system [130]. Several studies have studied and supported the concept of NS’s anti-Alzheimer’s action.

Recent evidence from Elibol et al. demonstrates that micro-osmotic pumps carrying Aβ1-42 may be successfully cannulated into the hippocampus area of adult female rats, where the impact of TQ can then be tested. After eliminating A plaques and restoring neuron viability, the researchers found that TQ (10 and 20 mg/kg) was effective against AD and led to improved memory [131]. TQ was found to be effective against A deposition and its neurotoxicity in human iPSC-derived cholinergic neurons and LPS/IFN-gamma-activated BV2 microglia cells by increasing antioxidant levels and decreasing synaptic plasticity and pro-inflammatory mediators by modulating NF-kappa B (NF-kb)-mediated signaling molecules [132,133]. When injected intraperitoneally at doses of 5 and 10 mg/kg for 4 weeks, TQ was similarly shown to significantly restore memory and learning functioning in male Wistar rats [134]. It has also been shown that NS seeds’ aqueous and methanolic extracts have a potent central nervous system depressant and analgesic effect [135]. The antioxidant and free radical scavenging characteristics of NS, which have been shown to improve memory, are likely attributable to the presence of one or more of its components [136]. It has been demonstrated that NS oil prevents lipid peroxidation in the hippocampus of rats subjected to ischemia-reperfusion damage [137]. Studies have shown that a component of NS is neuroprotective, and this effect has been demonstrated in several disease states, including type 2 diabetes. In most cases of neural dysfunction, an active insulin signaling cascade is neuroprotective. The oxidative stress, pro-inflammatory mediators, and amyloidogenic pathway are all modulated by NSO therapy in a model of Alzheimer’s disease in type 2 diabetic rats. Intriguingly, scientists have also examined the brain’s miRNA pool following NSO therapy, revealing a return to pre-AD levels [138].

### 5.7. Obesity

Several factors have contributed to the alarming rise of obesity in recent decades [139]. The World Obesity Atlas 2022, published by the World Obesity Federation, predicts that one billion people globally, including 1 in 5 women and 1 in 7 men, will be living with obesity by 2030. The findings highlight that countries will not only miss the 2025 WHO target to halt the rise in obesity at 2010 levels, but that the number of people with obesity is on course to double across the globe [140]. Obesity has been linked to an increased risk of many chronic illnesses, including CVDs, atherosclerosis, type 2 diabetes, various forms of cancer, and non-alcoholic fatty liver disease [141]. Reducing caloric intake and increasing exercise are two of the cornerstones of obesity therapy, along with the use of anti-obesity drugs. However, synthetic anti-obesity drugs have adverse side effects, and their effectiveness frequently wanes with continued usage [142]. The public and scientific community have recently shown much interest in plant-based nutrition and medicinal plants as potential alternatives to conventional medicine. Compared to synthetic medications, medicinal plants are easier to get, cheaper, and have fewer adverse side effects [143]. Some medicinal plants have shown promise for treating obesity and related health issues [143,144]. NS, also known as black seed, is one of these herbs [111,145,146,147].

**Figure 7:**
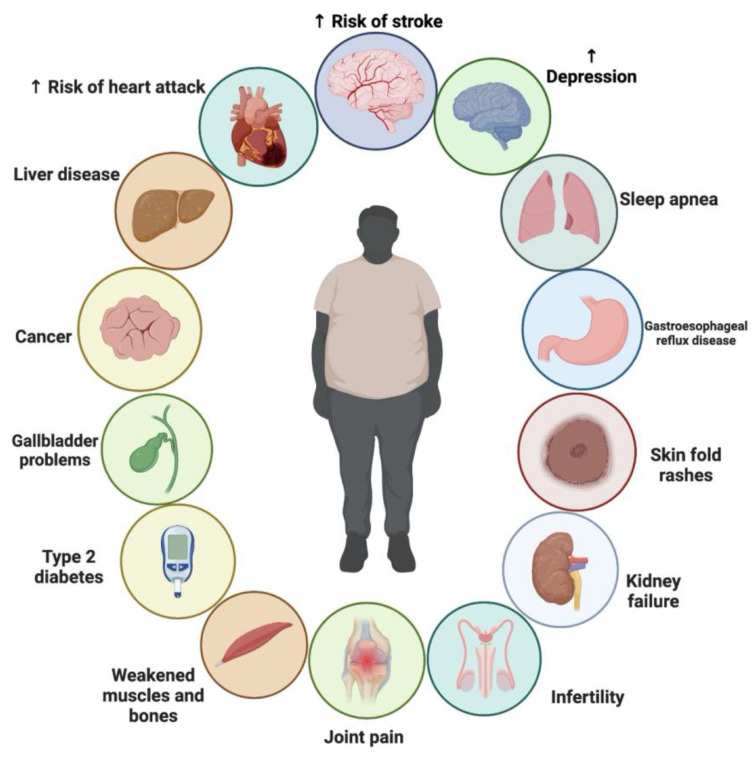
Chronic problems associated with obesity.

There is ongoing debate about whether NS is effective as an adjunct treatment for weight loss. TQ, which comprises up to 30–48% of NS oil, has been linked to medicinal benefits, including its ability to reduce body fat, which was discovered in recent studies. The pharmacological effects of NS may also be due to other compounds found in the plant, such as thymohydroquinone, thymol, thymoquinone, nigellone and its derivatives; nigellone and its derivatives; nigellone and its derivatives; fatty acids; and flavonoids [148]. The consumption of NS was shown to reduce food intake and enhance energy expenditure in animal models [149,150,151]. In addition, the plant showed no adverse severe or toxicological effects in either human or animal studies [147,149,152,153]. While NS has been shown to help with weight control in certain studies, others have shown little to no benefit [9,147,154,155,156,157,158]. Therefore, the evidence for suggesting NS as a weight-loss supplement is still lacking. Various clinical trials showed that the supplementation with NS exerted moderate effects as a complementary therapy for reducing body weight. No severe side effects were also reported following NS supplementation. Figure 7 shows the chronic problems associated with obesity.

### 5.8. The Influence of NS on Asthma Control

Inflammatory mediators, chronic inflammatory reactions, and oxidative stress play a central role in the pathogenesis of many lung disorders, such as tracheitis, chronic obstructive pulmonary diseases, and asthma [159]. The prevalence of asthma ranges from 1% to 18% worldwide, making it a severe public health concern [160,161]. The symptoms result from a persistent inflammatory process that is mediated and coordinated by the byproducts of specific immune cells [162,163]. However, no effective asthma preventive measures or cures have been discovered, and current treatment focuses on achieving and maintaining clinical control. These strategies may not prevent asthma-related chronic inflammation and remodeling [164,165].

Herbal remedies for asthma in humans and animals have come a long way in recent years [166]. For over 2000 years, NS has been used to treat various illnesses. Research on humans and animals has suggested that NS may have anti-asthmatic properties [93,167,168]. NS has healing and protective properties, such as bronchodilation, reducing inflammation, and protecting against allergies. It also works well at changing the immune system to treat many lung diseases [169,170,171]. It can inhibit histamine receptors, has anti-cholinergic properties, and relaxes various smooth muscle preparations [172,173,174]. In earlier research, NS has been shown to have immunomodulatory, anti-inflammatory, and antioxidant properties [68]. NS has been studied in limited clinical trials for its potential in alleviating asthma symptoms, with results showing substantial improvements in subjective well-being and pulmonary function [175,176,177]. Supplementation with NS for asthmatic patients is not yet supported by enough data to be used routinely in clinical practice. NS supplementation for these individuals has been the subject of numerous recent research, with mixed outcomes [178]. There needs to be more in vivo research showing the effects of NS oil on animals and people [179]. The authors investigated the effects of NS oil in an experimental model of allergic airway inflammation in rats. Oil was injected intraperitoneally before exposing rats to ovalbumin (OVA). The authors examined spleen T cell proliferation and evaluated total IgG1, IgE, and OVA-specific IgG1 blood levels. In addition, they analyzed the expression of genes encoding various cytokines, such as IL-4, IL-5, IL-6, and transforming growth factor-1 (TGF-1). NS oil inhibited inflammatory cell infiltration and pathological lung lesion development, demonstrating its ability to dampen Th2-type responses in rats. NS oil treatment also inhibited T-cell proliferation in the spleen [179]. Balaha and his colleagues studied how giving NS oil to a mouse model of allergic asthma could reduce inflammation and change the immune system. Oral administration of 4 mL/kg/day NS oil dramatically reduced airway responsiveness in OVA-sensitized mice [168].

Numerous clinical research indicated that NS extract has bronchodilator, preventative, and curative therapeutic effects on individuals with chronic asthma, leading to a substantial reduction in blood biomarkers of asthma and an improvement in asthmatic symptoms. It also resulted in a 5–75% improvement in peak expiratory flow. The inhibition of histamine release and practical anti-inflammatory effects on mast cells after treatment with NS formulations resulted in lower fractional exhaled NO and serum IgE levels, respectively [170,171,180]. There is a balance between Th1 and Th2 cytokines, thanks to the activation of immune cells and the initiation of the antigen presentation system by black cumin seed or its active compounds. The bioactive compounds in black cumin seeds make it easier for LC3II to turn into LC3III, which is a sign that autophagy is starting to work. Black cumin improves anti-histamine, anti-inflammatory-GPx, and SOD-responses, while reducing inflammation and oxidative stress [181]. These results suggest that oral supplementation with NS may be helpful in the treatment of bronchial asthma.

### 5.9. Nigella sativa for the Treatment of COVID-19

The first identification of Coronavirus disease-2019 (COVID-19) occurred in China in December 2019. This illness’s causative agent has been Severe Acute Respiratory Syndrome Coronavirus-2 (SARS-CoV-2). SARS-CoV-2 has since disseminated globally, precipitating a substantial epidemic and posing significant obstacles to global healthcare systems [182,183,184]. The majority of individuals inflicted with COVID-19 exhibit either no symptoms or a mild illness that can typically be controlled with the administration of antipyretics, analgesics, hydration, and the application of constitutional symptoms; clinical deterioration is closely monitored [185]. In light of the ongoing epidemic, the quest for a viable therapeutic intervention has emerged as a paramount focus within scientific medical investigation [186].

**Figure 8:**
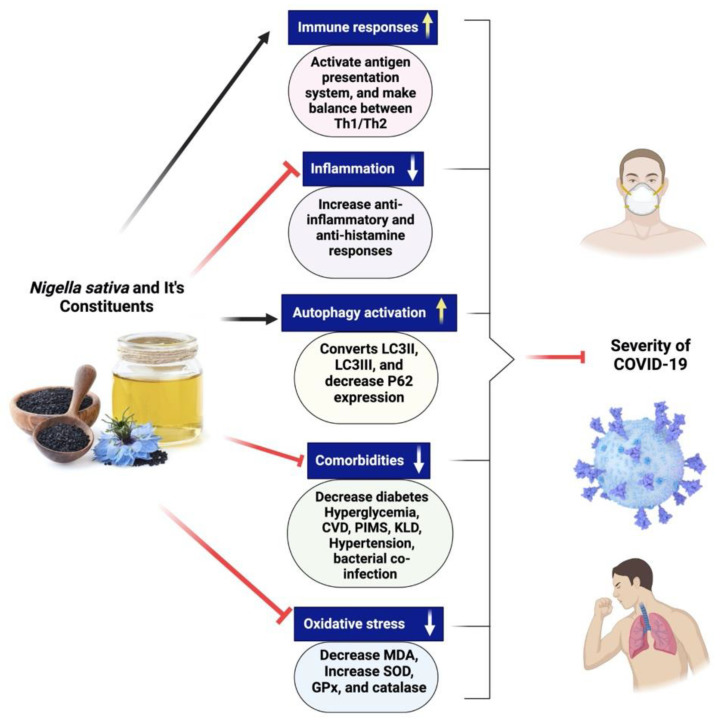
Different mechanisms of action of *Nigella sativa* and its secondary metabolites for COVID-19. NS seeds and its active constituents activate immune cells, make balance between Th1 and Th2 cytokines and initiate antigen presentation system, decreases inflammation, increases anti-inflammation and antihistamine responses. Black cumin bioactive compounds increase autophagy activation by the conversion of LC3II to LC3III, improving comorbidities situation in SARS-CoV-2-infected patients and help in decreasing oxidative stress.

There are not currently many pharmacotherapeutic medications available that work against COVID-19. Hence, the potential use of complementary herbal medicines, which possess diverse biologically active compounds, is being explored as a therapeutic approach for combating coronavirus infection [81,160,166]. These therapeutic interventions are often used to treat respiratory disorders, and reports have shown their effectiveness in alleviating influenza-related symptoms [187]. *Nigella sativa* has been recognized as a promising phytomedicine owing to its diverse pharmacological properties, including antiviral, anti-inflammatory, and immunomodulatory actions [188,189]. NS is a well-known cooking spice that has significant health benefits and an extensive history [7,187]. Numerous bioactive constituents have been discovered inside NS, among which thymoquinone is a prominent component [7]. Thymoquinone safety profile has been the subject of multiple clinical studies, one of which involved asthmatic patients and was completed by us [190]. Many preclinical and clinical investigations have provided evidence of NS’s antiviral properties against various viruses [191,192,193,194,195,196,197]. Research conducted in vitro demonstrated a reduction in the viral load of the coronavirus when exposed to NS [197]. Certain compounds derived from NS have shown promising inhibitory effects on the propagation of coronaviruses in computational models [198]. Several chemical compounds present in NS, including α-herein, nigellidine, hederagenin, thymoquinone, and thymohydroquinone, have shown promise as active molecules that might suppress the coronavirus [198]. There is a need to do more preclinical studies to establish that NS might be fighting against coronavirus. A clinical study of NS in patients with COVID-19 is recommended to investigate its clinical efficacy if preclinical research provides positive results. Figure 8 exhibits the different mechanisms of action of *Nigella sativa* and its secondary metabolites for COVID-19.

## 6. Clinical Trials

Clinical trials are studies that evaluate the effects of treatment on humans, whether pharmaceutical, behavioral, or surgical. Clinical trials are the gold standard for testing the efficacy and safety of potential new treatments and preventative measures in humans. New illness detection, diagnosis, and treatment methods are tested in clinical trials. Randomized controlled trials (RCT) are a kind of scientific experiment that aim to reduce bias when testing new interventions. Trial participants are randomly allocated either to the group receiving the treatment under investigation or to a control group receiving standard treatment (or a placebo) [199]. A clinical study was conducted to examine the effects of NS oil on the lipid profile, fasting blood glucose (FBG), and systemic inflammation in patients with (T2DM). Fifty type 2 diabetes patients were used in this randomized, double-blind clinical investigation. Patients were randomized to receive either an oil extracted from NS or a placebo. Results of the clinical study have shown that patients with T2DM who take an NS oil supplement see improvements in their lipid profiles and glycemia, as well as decreases in their C-reactive protein levels and lipid peroxidation [200]. Another trial was conducted to identify the possible blood glucose-lowering effects of the black seed oil on healthy subjects. Neither group had severe adverse effects on their livers, kidneys, or intestines. Giving 5 mL of black seed oil to healthy people every day for two months improved their glycemic profile without causing other problems [201]. NS and its secondary metabolites are used as antioxidants and anti-inflammatories in medical sciences. An examination was conducted to determine the effects of different fasting blood sugar levels and lipid profiles in nonalcoholic fatty liver disease (NAFLD) patients via a clinical study. Results showed that NS seed oil supplementation led to lower lipid profiles in the FBS [202].

Razmpoosh et al. conducted a clinical trial to determine the effect of NS oil supplements on CVD risk factors. Obese and overweight healthy women were randomized to receive NS oil (2000 mg/day) and placebo. This intervention period lasted eight weeks and was separated by a 4-week washout period. Overweight and obese women were randomized to receive 2000 mg/day of NS oil and placebo for eight weeks and separated for a 4-week washout period. The overall reductions in CVD risk factors demonstrated the advantageous effects of NS supplements in preventing potential cardiovascular diseases in adults who are obese [203]. NS seed oil was studied to see whether it could be used as a supplemental therapy for hypertension, glycemic control, and lipid metabolism, and if it was safe to do so. Patients with hypertension were randomly assigned to either the intervention (*n* = 26) or placebo (*n* = 29) groups, where they were given 2.5 mL of oil extracted from NS seeds twice daily for 8 weeks or sunflower oil. Supplementing with NS seed oil showed further antihypertensive benefits in hypertensive patients, with no adverse events recorded in the kidneys, the liver, or by the patients themselves, and positive effects on glucose control and lipid metabolism [204]. The risk factors for cardiovascular disorders are strongly associated with NAFLD. The study aimed to assess how supplementing with black seed affected the risk factors for cardiovascular diseases in NAFLD patients. Fifty people were included in this placebo-controlled, randomized clinical study of NAFLD treatment. For 12 weeks, participants were given either 2 g of NS or a placebo and advice on improving their lifestyle. The results show that patients with NAFLD benefit more from a combination of lifestyle changes and daily ingestion of 2 g NS than from lifestyle changes alone [205].

To find out the efficacy of NS in central obese men on waist circumference, body weight, blood sugar, lipid, adiponectin, hs-CRP Test (C-Reactive Protein High-Sensitivity), uric acid, and side effects in the treatment group compared to control. Participants aged 30 to 45 are randomly assigned to either a treatment or a control group and monitored weekly for three months. Results have shown that both body mass index and waist size decreased significantly, and that there was no significant effect on systolic and diastolic blood pressure. The therapy group showed no signs of adverse effects [205]. Dietary changes can effectively manage and prevent hypertension (HT), a disease associated with lifestyle choices. A double-blind, randomized, placebo-controlled study was conducted to assess the effectiveness of oral NS seed extract supplementation as a treatment for patients with mild HT. Randomization assigned participants to receive either a placebo or 100 or 200 mg of NS extract twice daily. After 8 weeks, the systolic blood pressure (SBP) levels in both case groups were much lower than at the beginning of the study. The two case groups’ SBP decrease was statistically significant compared to the placebo group. The findings indicate that moderate HT patients who take NS seed extract daily for 2 months may see a reduction in blood pressure [206].

Kalonji seed in capsules was examined for its impact on serum lipid levels, blood pressure, body weight, and blood sugar in adults. Sixty-four patients were assigned to the treatment group, while fifty-nine were assigned to the control group. Nearly all variables showed a positive effect from powdered Kalonji seed in capsule form. However, the small sample size prevented the results from being statistically significant [207]. Randomized and single-blind research examined how NS affects healthy senior participants’ memory, attention, and cognition. NS’s safety profile was also evaluated during the study’s nine weeks. Forty senior citizens were enlisted and split evenly into groups (A and B), each with 20 participants. Group A was given 500 mg of NS in capsule form twice daily for nine weeks, whereas Group B was given a placebo instead of NS. The results of this research support the use of NS for improving learning, recall, and reasoning. Therefore, whether NS could be considered as a potential food supplement for preventing or slowing the progression of Alzheimer’s disease needs further investigation. Before using NS daily, large-scale cohort studies with Alzheimer’s patients are recommended, as are phytochemical studies for novel NS drugs for cognitive disorders [208].

People who are overweight or obese usually have higher risk factors for CVDs. As a result, a therapeutic approach aimed at controlling metabolic profile and body weight may effectively prevent CVDs. In a double-blind, randomized, controlled clinical trial, 90 obese women investigated the effects of NS oil and a low-calorie diet on obese women’s cardiometabolic risk factors. All of the women in the study ranged in age from 25 to 50, and their body mass index (BMI) was between 30 and 35 kg/m^2^. For eight weeks, they were randomized to either a placebo or a low-calorie diet supplemented with 3 g (1 g before each meal) of NS oil daily. A total of 84 women participated in the study (43 in the intervention group and 41 in the placebo group). The fundamental features of the two groups were comparable. Both groups altered their diets after the intervention compared to before it, although the changes were not statistically different. More research is required to determine whether or not NS is effective as an adjunct treatment for obesity [209]. 50 volunteer obese women aged 25–50 years with 30–35 kg/m^2^ were recruited in a double-blind, placebo-controlled randomized clinical trial. When used with a calorie-restricted diet, NS oil led to more significant weight loss in the NS group than in the placebo group. Compared to the placebo group, those using NS oil lost more weight while on a low-calorie diet. Weight loss and elevated superoxidase dismutase (SOD) levels occurred simultaneously in obese individuals who used NS oil in conjunction with a reduced-calorie diet [210].

Khonche et al. conducted a randomized, double-blind parallel control study to treat patients with NAFLD. Every twelve hours, sixty patients got 2.5 mL of fully standardized NS seed oil, and the other sixty patients received a placebo for three months. Outcomes have shown that NS seed oil seems safe and improves liver steatosis, LDL-C, HDL-C, and injury in NAFLD patients [211]. The NS intervention’s effects on weight, height, waist circumference, and other body measurements, as well as food consumption and satiety, were studied in a randomized, double-blind, crossover clinical study. Forty-five healthy women who were overweight or obese were randomized into two groups-intervention and placebo-the research was carried out throughout two 8-week intervention periods, with a 4-week washout period. Dietary consumption and anthropometric parameters were also assessed. The good effects of NS supplements in treating obesity are shown by overall improvements in body composition and anthropometric parameters and a significant decrease in appetite [212]. Table 1 shows the pharmacological effects of *Nigella sativa*.

## 7. Toxicological Properties

The seeds of the NS plant contain a wealth of beneficial elements. Several hepatic and renal enzymes and metabolites were unaffected by 50 mg/kg intraperitoneal NS seed extract in rats for five days [213]. Rats and mice given the seed oil orally at dosages up to 10 mL/kg showed no signs of death or overt toxicity after 48 h [214]. It was verified in a study, which found that giving rats a dosage of 10 mL/kg of fixed oil of NS orally for up to 12 weeks did not result in any deaths or appreciable changes to their vital liver enzymes [149]. It was determined that the LD_50_ value of thymoquinone is 2.4 g/kg (range 1.52–3.77) [215]. For ninety days, mice given up to 0.03% of thymoquinone in their drinking water showed no toxicity, except for a notable drop in fasting plasma glucose concentration [215]. There was no evidence of harm when thymoquinone (at concentrations up to 0.03%) was added to the drinking water of mice for 90 days. However, there was a considerable reduction in fasting plasma glucose content. Topical corticosteroids were effective in treating several conditions [216]. To evaluate the safety of NS as an herbal remedy, healthy volunteers were administered 5 mL/day of NS oil for eight weeks. No significant adverse effects were observed on the kidney, gastrointestinal tract, or liver [217]. An additional clinical trial involving 39 obese men demonstrated that consuming NS seeds (3 mg daily for three months) had no discernible adverse effects [152]. Clinical investigations using NS seeds found no significant impact on blood creatinine or alanine aminotransferase (ALT) levels in people when given 2 g/day for 6 weeks [207].

Moreover, there was no discernible change in the renal or hepatic function of diabetic patients who took NS at doses of 1, 2, or 3 g/day for three months [218]. Patients with seasonal allergy rhinitis (AR) participated in a clinical experiment to assess the safety of NS consumption. Patients were instructed to take 250 mg daily for two weeks, after which they were to report any adverse effects. Furthermore, it was verified that nasal dryness was observed in patients treated with nasal drops containing NS oil for seasonal AR [219]. Both aspartate transaminase (AST) and alanine transaminase (ALT) enzyme levels were significantly increased only from the oil.

Contrarily, when crushed seeds were combined with the oil, the alkaline phosphate and γ-GT activities substantially increased. Therefore, NS is considered safe for therapeutic and dietary purposes [156]. Combining the usual diabetes treatment (medication, exercise, and a healthy diet) with NS tea (5 g/day) was the subject of a clinical investigation. For two months, patients with mild hypertension who took 200 or 400 mg/day of NS seed extract did not experience any significant side effects [206]. The findings showed that patients with type 2 diabetes had a realistic liver and kidney safety level. The overall number of leukocytes and platelets was unaffected by the consumption of NS oil [220]. Another clinical experiment investigated the potential for NS to have adverse effects or safety when used to treat childhood cancer. In addition to the standard chemotherapy, NS powder is administered to children aged 2–18 with acute lymphoblastic leukaemia at 40 mg/kg divided into two doses over three months. NS had fewer side effects than L-asparaginase and standard drugs such as prednisolone, daunorubicin, and vincristine [221]. These clinical results show that NS has the potential to be an effective natural anticancer medication and a vital alternative to L-asparaginase in the treatment of cancer-affected individuals.

In addition, no adverse side effects were reported when children infected with cestodes were given NS seeds as part of a clinical experiment looking at the seeds’ anticestodal benefits [222]. Another research showed that interference with other cytochromes’ substrates, including CYP2D6 and CYP3A4, led to a reduction in the absorption of dextromethorphan when NS (5 g/day) was used by healthy human volunteers [223]. NS may also impact the metabolism of numerous other medications, therefore care should be exercised [224]. In addition to its many positive functions and effects on biological systems, NS has been extensively studied to ascertain its toxicity. One of these studies was done to determine if NS seeds could harm liver tissue and enzymes. Serum levels of ALT and AST were not significantly altered by NS therapy. Researchers discovered that it mitigated toxicity by functioning as an antitoxic. According to the results, it is safe for living organisms [225,226]. Animal studies have shown that high doses of NS may harm the kidneys and liver. Taking NS while undergoing chemotherapy may also reduce the effectiveness of the medications. Nevertheless, prolonged exposure to NS extracts may result in their toxicity to biological systems [4].

As a result of the numerous studies conducted to determine the safety of NS, its use in all biological systems is deemed relatively risk-free. In food and medicine, black seed is safe when used in modest doses for a short time. However, it harms living organisms when used in high quantities or over extended periods. Black seed, such as culinary seasoning, is probably safe for most people to eat in small amounts. When taken in small doses for a limited period for medical purposes, black seed oil and extract may be safe. Not enough information is available to determine whether more significant quantities, such as those used in medicine, are safe.

## 8. Conclusions and Future Recommendations

Anti-inflammatory medicinal herbs have shown beneficial effects in fighting inflammatory responses. A literature survey has demonstrated that NS has protective effects in inflammatory diseases. We discussed the examination of NS’s anti-inflammatory properties in both experimental and clinical settings, with the findings from clinical settings being the more trustworthy. In vitro and in vivo studies have shown impressive pharmacological potential against chronic inflammatory diseases due to their high concentrations of TQ, α-hederin, and oil extracts in NS. The anti-inflammatory qualities, widespread availability, affordability, sufficient potency, little or absent side effects, and enhanced safety and efficacy compared to synthetic alternatives make NS and its phytoconstituents advantageous. There are various mechanisms for the anti-inflammatory action of these secondary metabolites. The potential exists for the synergistic modulation of factors, enzymes, and proteins involved in the anti-inflammatory pathway and the potential for interference with these components within the inflammatory pathway. Such factors include interleukins, tumor necrosis, cyclooxygenases, lipooxygenases, nuclear factors, mitogen-activated protein, nitric oxide, prostaglandin, and others. The research suggests NS may be a potential anti-inflammatory drug for cardiovascular, diabetes, and asthma-related conditions. To date, clinical trials conducted with NS are limited. More clinical research with more participants and a meta-analysis might help resolve some disagreements. Further investigation is required to ascertain the optimal dose and duration of treatment necessary to establish the effectiveness and use of *Nigella sativa* in managing chronic inflammatory conditions.

## Figures and Tables

**Table 1:** Summary of clinical trials showing the pharmacological effect of *Nigella sativa*.

Participants	Interventions	Comparisons	Outcomes	Study Design	References
Fifty type 2 diabetes patients	Two capsules of 1000 mg *N. sativa* oil were given daily to the intervention group for eight weeks.	Randomly assigned to intervention and placebo	NS oil supplements see improvements in their lipid profiles and glycemia.	RCT	[200]
Seventy healthy subjects	2.5 mL Black seed oil, two times a day.	NS oil vs. placebo (mineral oil)	Significant decrease in HbA1c levels and fasting blood glucose.	RCT	[201]
Forty-four patients with NAFLD	Once a day, 1 g of NS oil orally for 8 weeks.	NS oil vs. placebo (paraffin oil)	Supplementing with NS seed oil improved lipid profiles and reduced FBS.	RCT	[202]
Thirty-nine patients with obese and overweight healthy women	2000 mg/day of NS oil for eight weeks	NS oil vs. placebo	Reductions in CVD risk factors	RCT	[203]
Fifty-five with hypertension	2.5 mL/day of NS oil for eight weeks	NS oil vs. placebo	Positive effects on glucose control and lipid metabolism	RCT	[204]
Fifty patients with NAFLD.	2 g NS plus lifestyle modification	NS or placebo	Improve insulin resistance	RCT	[205]
One hundred and twenty-three	Kalonji seed in capsules form	Kalonji seed vs. placebo	Positive effects on FBS, serum lipids.	RA/DB/parallel	[207]
90 obese women	3 g (1 g before each meal) of NS oil daily	NS oil vs. placebo	Weight and waist circumference decreased significantly.	RA/DB/parallel	[209]
50 obese women	a low-calorie diet with 3 g/day NS oil	NS oil vs. placebo	Weight loss and elevated superoxidase dismutase (SOD) levels.	RA/DB/parallel	[210]
Forty-five healthy women with obesity	2000 mg of NS oil with supplement	NS oil vs. placebo	Good effects of NS supplements in treating obesity.	RA/DB/crossover design	[212]
80 asthmatics patients	NS oil capsules 500 mg twice daily for 4 weeks	NS oil vs. placebo	NS oil supplementation, asthma control and pulmonary function improvement.	RA/DB/placebo-controlled trial	[190]

Non-alcoholic fatty liver disease (NAFLD); fasting blood sugar (FBS); cardiovascular disease (CVD); Randomized (RA); Double-blinded (DB); Randomized controlled trials (RCT); superoxidase dismutase (SOD).
